# Non‐Melanoma Skin Cancer in Transplant Recipients: A Single‐Centre Retrospective Cohort Study on the Role of Transplant Type, Immunosuppressive Exposure, and Tumor Subtypes

**DOI:** 10.1111/ctr.70451

**Published:** 2026-01-30

**Authors:** Corrado Zengarini, Vittoria Tagnin, Gioia Sorbi, Alba Guglielmo, Michelangelo La Placa, Bianca Maria Piraccini, Alessandro Pileri, Marco Pignatti

**Affiliations:** ^1^ Department of Medical and Surgical Sciences University of Bologna Bologna Italy; ^2^ Dermatology Unit IRCCS Azienda Ospedaliero‐Universitaria Di Bologna Bologna Italy; ^3^ Plastic Surgery Unit IRCCS Azienda Ospedaliero‐Universitaria Di Bologna Bologna Italy; ^4^ Department of Medical Sciences Section of Dermatology and Infectious Diseases University of Ferrara Ferrara Italy

**Keywords:** BCC, immunosuppression, long‐term follow‐up, malignancies, NMSC, organ transplantation, SCC, survival analysis, skin cancer screening, transplant recipients

## Abstract

**Background:**

Non‐melanoma skin cancer (NMSC) is a frequent long‐term complication in transplant recipients, mainly due to chronic immunosuppression. Its incidence varies by transplant type and regimen, but existing evidence is often fragmented.

**Objective:**

To assess long‐term incidence and risk factors for NMSC, comparing transplant types, immunosuppressive regimens, and tumor subtypes.

**Methods:**

This retrospective cohort comprised 901 transplant recipients (1975–2024) who were under long‐term dermatologic follow‐up. Data on transplant type, immunosuppressive regimen/duration, and tumor subtype were analyzed. NMSC‐free survival was evaluated with Kaplan–Meier curves; Cox regression assessed predictors.

**Results:**

Among 901 transplant recipients, 191 (21.2%) developed at least one NMSC during long‐term follow‐up. Crude incidence rates ranged between 1.88 and 2.68 per 100 person‐years across immunosuppressive drug classes and agents. In multivariable Cox models, older age at first transplant was independently associated with higher NMSC risk (HR 1.07 per year, 95% CI 1.06–1.09, *p* < 0.001). Sex, transplant group, and ever‐use of individual immunosuppressive agents were not significantly associated with NMSC risk after adjustment.

**Conclusion:**

In this long‐term cohort, NMSC risk increased with age at first transplant, whereas transplant organ and ever‐use of major immunosuppressive agents were not independently associated in adjusted models. Over an extended follow‐up, NMSC accumulated steadily, with crude incidence rates around 1.88–2.68 cases per 100 person‐years across immunosuppressive classes and agents, underscoring the cumulative nature of skin cancer risk under chronic immunosuppression. Our results reinforce the need for sustained, long‐term dermatologic surveillance, particularly in older patients undergoing transplantation.

## 1 Introduction

Cutaneous malignancies encompass both the aggressive and potentially fatal melanomas and the more common non‐melanoma skin cancers (NMSC). While NMSCs, including basal cell carcinoma (BCC) and squamous cell carcinoma (SCC), generally have lower metastatic potential than melanomas, their clinical management can pose significant challenges. This is particularly true in immunocompromised patients, such as organ transplant recipients, where the interplay of chronic immunosuppression, increased tumor burden, and higher recurrence rates necessitates a multidisciplinary and individualized approach [1, 2, 3, 4, 5]. These complexities underline the importance of tailoring diagnostic and therapeutic strategies to address the unique risks and needs of this patient population.

A key distinction between NMSC and melanoma lies in the underlying molecular and environmental drivers of tumorigenesis. While melanoma is often characterized by mutations in key regulatory proteins such as BRAF, NRAS, and KIT, driving aberrant MAPK signaling, the pathogenesis of NMSC is equally complex but distinct [6, 7, 8]. In NMSC, particularly squamous cell SCC and BCC, chronic UV radiation exposure induces a cascade of mutational events, including hallmark “UV signature” mutations, primarily involving pyrimidine dimers and mutations in tumor suppressor genes such as TP53 and PTCH1 [9, 10, 11].

Beyond UV‐induced genetic alterations, the development of NMSC is closely linked to chronic inflammation and impaired immune surveillance. Calcineurin (CNI) inhibitors and azathioprine (AZA) not only suppress T‐cell responses but also amplify UV‐related DNA damage and hinder DNA repair [12]. Pro‐inflammatory cytokines like IL‐6 and TNF‐α further promote a tumor‐supportive microenvironment. Prolonged immunosuppression, therefore, allows atypical keratinocytes to accumulate and expand, leading to multiple, locally aggressive, and sometimes metastatic tumors. The magnitude of this risk varies across transplant types: SOTRs show a 65‐ to 250‐fold increase in cutaneous SCC compared with the general population [4, 13, 14], reflecting chronic lifelong pharmacologic immunosuppression, whereas HSCT recipients have a lower but still elevated risk [1, 15], mainly modulated by graft‐versus‐host disease and its treatment [16, 17, 18, 19]. While this inflammatory environment can contribute to carcinogenesis, the overall lower and time‐limited exposure to systemic immunosuppression may help explain the comparatively reduced incidence of NMSC in this group [4, 20, 21].

Given this elevated risk, multiple guidelines advocate for regular dermatological surveillance in transplant recipients, emphasizing early detection and management of NMSC [2, 22]. However, existing data can sometimes be discordant, reflecting variability in patient demographics, types of immunosuppressive regimens, therapy duration, and the type of transplant performed [23]. While younger recipients may benefit from prolonged post‐transplant follow‐up, older patients often present with pre‐existing UV‐induced skin damage, further complicating the risk profile [19, 20, 24].

In this retrospective study conducted at the IRCCS Azienda Ospedaliero‐Universitaria di Bologna (Bologna, Italy), within the dermatological screening and surveillance service for immunosuppressed patients, in collaboration with Plastic Surgery, we aimed to analyze the incidence and characteristics of NMSC across different transplant recipients, exploring how variables such as transplant type, immunosuppressive protocols, and baseline patient factors influence outcomes. This work seeks to comprehensively understand NMSC risk in this vulnerable population and inform tailored surveillance strategies to optimize dermatological care.

## 2 Materials and Methods

### 2.1 Data Collection

Clinical records (electronic and paper charts) of transplant recipients followed at the dermatologic surveillance service jointly run by the Dermatology Unit and the Plastic Surgery Unit of the IRCCS Azienda Ospedaliero‐Universitaria di Bologna were retrospectively reviewed between January 1975 and April 2024. Collected data included demographics (age, sex), transplant details (organ type, time since transplant), and dermatologic findings. Histopathological confirmation was obtained when indicated, with documentation of lesion type, location, and characteristics.

The following variables were considered: patient demographics, organ transplanted, time since transplantation (immunosuppressive regimen), number and type of NMSC (BCC, SCC, or both), and time to first skin cancer diagnosis, confirmed by histopathology (including diagnosis, margins, and invasion depth when available). The primary endpoint was time from first transplant to the first histologically confirmed NMSC. Patients without NMSC were censored at the date of last dermatologic follow‐up.

Ethical approval for this retrospective study was obtained in the first quarter of 2019 from the local Ethics Committee AVEC (Bologna, Italy) (approval code: Clin.Isto.Tp.19).

### 2.2 Immunosuppressive Exposure

Immunosuppression was analyzed by class (calcineurin inhibitors, antimetabolites, mTOR inhibitors, and corticosteroids) and by individual agents. For each class and agent, ever‐use was coded as exposure at any time during follow‐up (switches and combinations allowed).

Cumulative time on immunosuppression, defined as the interval from transplant to last follow‐up, was used only to compute person‐year incidence rates. Because this variable was highly collinear with follow‐up time, it was not included as a fixed covariate in Cox models.

### 2.3 Transplant Grouping

Transplants were grouped into five categories: heart, combined/multiorgan transplants, HSCT, liver, lung, and kidney.

Descriptive statistics summarized baseline and clinical characteristics (means/SD or medians for continuous variables; counts and percentages for categorical variables). NMSC‐free survival was estimated with Kaplan–Meier curves and compared between groups using log‐rank tests. Cox proportional hazards models assessed predictors; proportional hazards were checked via Schoenfeld residuals. Two‐sided *p* < 0.05 was considered significant. Analyses were run in SPSS v26 and Python (lifelines/Matplotlib).

## 3 Results

Of the 1,005 patients initially screened, 901 met the inclusion criteria. The earliest transplant in this series dates back to 1975, and the most recent to 2024. The cohort consisted of 590 males (65.5%) and 311 females (34.5%).

The majority of patients were of Italian nationality, accounting for 64.9% of the cohort, followed by individuals from other Southern European countries (5.2%), Eastern Europe (7.4%), North Africa and the Middle East (10.1%), Sub‐Saharan Africa (3.4%), South Asia (2.7%), East and Southeast Asia (2.2%), Latin America (2.3%), and Western or Northern Europe (1.7%). Correspondingly, the most common probable ethnic background was White Southern European (70.1%), followed by Middle Eastern/North African (10.1%), Eastern European (7.4%), Black Sub‐Saharan African (3.4%), South Asian (2.7%), Latino/Hispanic of mixed heritage (2.3%), East/Southeast Asian (2.2%), and White Northern/Western European (1.7%) (Table 1).

**TABLE 1:** This table presents the demographic and clinical data of transplant recipients, categorized by transplant type and grouped into five categories: Group 1 (heart transplants), Group 2 (combined transplants), Group 3 (liver OLT), Group 4 (lung transplants), and Group 5 (kidney transplants).

Category	Group	Total cases = 901		
**Ethnicity**		**Cases**	**Males**	**Females**
	**Caucasian** – Eastern Europe	67	37	30
	**Caucasian** – Italy	585	311	
	**Caucasian** – Southern Europe	47	23	24
	**Caucasian** – Western/northern Europe	15	6	9
	**East/Southeast Asia**	20	12	10
	**South Asia**	24	14	10
	**Latin America**	21	13	8
	**North Africa/Middle East**	91	51	40
	**Sub‐Saharan**	31	19	12
**Fizpatrick Scale**	**I**	36	17	19
	**II**	292	161	131
	**III**	356	183	173
	**IV**	144	84	60
	**V**	55	31	24
	**VI**	18	10	8
**Transplant type**	**Group**	**Total patients**	**Males**	**Females**
	Heart	121	96	25
	Heart and liver	2	2	0
	heart and kidney	2	2	0
	Liver and kidney	7	5	2
	Kidney and pancreas	12	4	8
	HSCT	81	49	32
	Liver	104	71	33
	Lung	54	23	31
	Kidney	518	338	180
**Age at first transplant**		**Mean age (years)**	**Median age (years)**	**Std Dev**	**Range**
	Heart	47.5	51	14.8	4 ‐ 67
	Heart and liver	37	37	22.6	21–53
	heart and kidney	49.5	49,5	7.7	44–55
	Liver and kidney	31.4	47	27.5	2–65
	Kidney and pancreas	40.2	40	10.1	24–54
	HSCT	47.3	49	14.9	16–70
	Liver	47.9	50	14.1	0 ‐ 74
	Lung	41.4	41.5	12.8	19 ‐ 65
	Kidney	46.1	48	15.6	0 ‐ 81
**NMSC frequencies by transplant type**		**Total cases (% on the group)**	**SCC cases**	**BCC cases**	**Mixed cases**	*p* Value
	Heart	31 (25.6%)	9	12	10	0.028
	Heart and liver	0 (0%)	0	0	0	1.000
	Heart and kidney	1 (50.0%)	1	0	0	0.780
	Liver and kidney	1 (14.3%)	0	1	0	1.000
	Kidney and pancreas	1 (8.3)	1	0	0	0.646
	HSCT	8 (9.9%)	2	6	0	0.080
	Liver	24 (23.1%)	5	12	7	
	Lung	3 (5.6)	0	2	1	0.028
	Kidney	122 (23.6%)	32	65	25	0.002
**Time to NMSC Development**		**Median (years)**	**Mean (years)**	**Std dev**	**Range**
	Heart	11.8	11	8.1	1.0–36.0
	Heart and Liver	0	0	N/A	N/A
	heart and Kidney	7	7		7.0–7.0
	Liver and Kidney	21	21		21.0–21.0
	Kidney and Pancreas	21	21		21.0–21.0
	HSCT	4.3	2.5	3.9	1.0–12.0
	Liver	12.5	12.5	7.5	1.0–31.0
	Lung	7	8	2.6	4.0–9.0
	Kidney	11.8	8	10.4	1.0–48.0
**Immunosuppressive therapy**		**CNI *n***	**Antimetabolites**	**mTORi *n***	**PDN *n***	**AZA**
	Heart	118	85	21	82	3
	Heart and liver	2	1	0	1	0
	heart and kidney	2	2	0	1	0
	Liver and kidney	7	4	1	5	0
	Kidney and pancreas	12	12	0	10	2
	HSCT	10	2	0	8	2
	Liver	95	24	10	23	2
	Lung	54	43	4	54	0
	Kidney	496	343	51	425	17

*Note:* For each transplant type, the total number of patients is reported along with the gender distribution (males and females). The age at first transplant is summarized with mean, median, standard deviation, and range. The occurrence of non‐melanoma skin cancer (NMSC) is detailed, including the total number of cases and the breakdown into squamous cell carcinoma (SCC), basal cell carcinoma (BCC), and mixed SCC + BCC cases. Additionally, the time to NMSC development is provided as a mean, median, standard deviation, and range. CNI exposure ranged from 91% to 100% in solid‐organ transplants (e.g., 95.8% kidney, 97.5% heart, and 100% lung), while it was 12.3% in HSCT. Antimetabolites were used by 70.2% (heart), 66.7% (lung), 57.3% (kidney), and 22.1% (OLT); mTORi exposure was less frequent but present across kidney (8.9%) and heart (17.4%) cohorts. Maintenance PDN ranged from 67.8% (heart) to 100% (lung) among solid organs and was 9.9% in HSCT.

The distribution of Fitzpatrick skin phototypes was as follows: phototype I in 36 patients, 17 males and 19 females; phototype II in 292 patients, 161 males and 131 females; phototype III in 356 patients, 183 males and 173 females; phototype IV in 144 patients, 84 males and 60 females; phototype V in 55 patients, 31 males and 24 females; and phototype VI in 18 patients, 15 males and 3 females. The mean age of the cohort was 58.17 ± 14.77 years, with a range from 17 to 91 years, consistent across phototypes and reflecting the characteristics of the long‐term post‐transplant follow‐up population (Table 1).

Among the 901 patients who had undergone transplantation and were followed up in the post‐transplant program:

‐ Kidney transplants: 518 patients (57.5%).
‐ Heart transplants: 121 patients (13.4%).
‐ Liver transplants (OLT): 104 patients (11.5%).
‐ Lung transplants: 54 patients (6.0%).
‐ Hematopoietic stem cell transplants (HSCT): 81 patients (9.0%).
‐ Combined transplants (e.g., heart and kidney): 23 patients (2.6%).

### 3.1 Mean Time Since Transplantation and Follow‐Up Age

The mean age of patients enrolled in the follow‐up protocol is of 58.17 years old, with a range spanning from 17 to 91 years old and a standard dev of 14.77. The mean age at the first transplant was 46.19 years (range: 0–81, SD: 15.32); this data was calculated considering the start of the immunosuppressive regimen. The highest mean age was observed in heart transplant recipients (47.55 years) and HSCT recipients (47.40 years), while the lowest mean age was in patients with combined transplants (38.09 years). If considered by each type of TR, the mean age was calculated as follows: heart transplants: 47.55 years (SD: 14.89); OLT: 47.96 years (SD: 14.17); lung transplants: 41.39 years (SD: 12.83); kidney transplants: 46.18 years (SD: 15.68); HSCT: 47.40 years (SD: 14.93); combined transplants: 38.09 years (SD: 17.69). data are summarized in Table 1.

### 3.2 Immunosuppressive Exposure and Regimens

Immunosuppression is described at the drug‐class level. For each class, including AZA, mycophenolate mofetil (MMF), CNI (tacrolimus, cyclosporine), mTOR inhibitors (sirolimus/everolimus), and maintenance corticosteroids, the exposure is captured using ever‐use indicators and cumulative years on drug.

The duration of immunosuppressive therapy was calculated as the time elapsed between the date of transplantation and the patient's age as of May 2024, which was the most recent update available in the clinical records.

The mean follow‐up time was 11.98 years (SD: 9.00, range: 0–49 years), with a median follow‐up of 8.0 years (IQR: 5–16 years). Follow‐up duration varied by transplant type, with heart and OLT recipients having the longest median follow‐up and HSCT recipients having the shortest.

The 901 transplant receivers (TRs) were classified based on their immunosuppressive therapy: 391 patients (43.4%) received triple immunosuppressive agents, which included a calcineurin inhibitor (ciclosporin or tacrolimus), an antimetabolite (azathioprine or mycophenolate mofetil), and prednisone, while 510 patients (56.6%) received non‐triple therapy, which involved other chronic immunosuppressive regimens.

CNIs were used by 796 patients (88.3%), comprising tacrolimus in 626 (69.5%) and cyclosporine in 174 (19.3%). Antimetabolites were used by 516 (57.3%), predominantly MMF (490, 54.4%), with AZA in 26 (2.9%). mTOR inhibitors were prescribed in 87 (9.7%), everolimus in 75 (8.3%), and sirolimus in 12 (1.3%). Maintenance corticosteroids (prednisone) were recorded in 609 (67.6%) recipients (percentages exceed 100% because of the combination regimens) (Table 1).

### 3.3 Prevalence of NMSC

**FIGURE 1:**
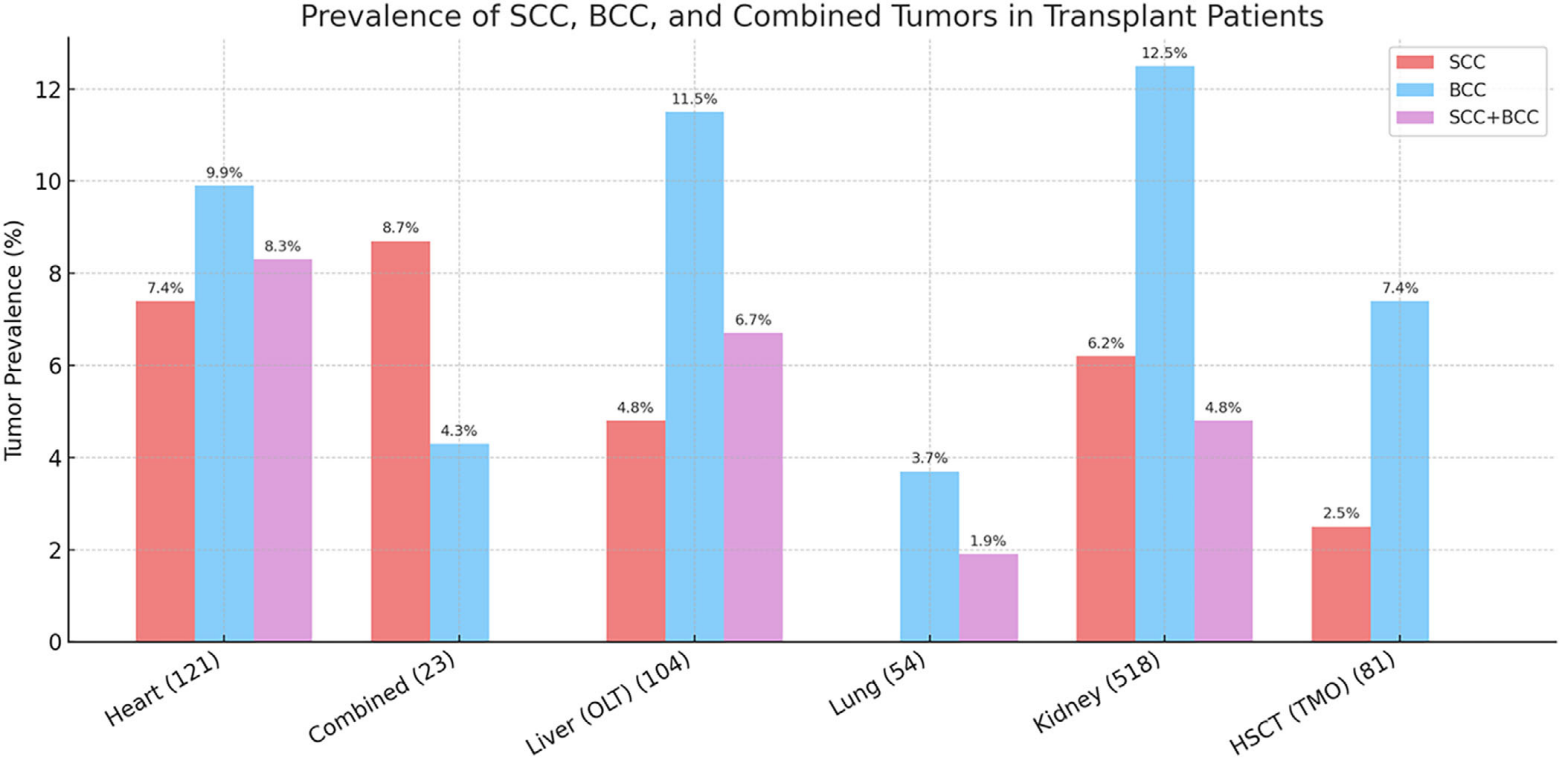
This bar chart illustrates the prevalence of non‐melanoma skin cancers (NMSC) among transplant recipients, categorized by transplant type. The three tumor types represented are squamous cell carcinoma (SCC, red), basal cell carcinoma (BCC, blue), and combined SCC + BCC cases (purple). Percentages indicate the proportion of patients within each transplant category who developed the respective tumor type. The data highlight variations in tumor prevalence across transplant types, with kidney transplant recipients exhibiting the highest percentage of BCC cases (12.5%), while heart and liver transplant recipients show lower prevalence rates. Lung and HSCT recipients have the lowest reported incidences. The differences in tumor burden likely reflect variations in immunosuppressive regimens, cumulative exposure, and patient‐specific risk factors.

Among 901 recipients, 191 (21.2%) developed at least one NMSC during follow‐up. Among NMSC cases, BCC accounted for 98/191 (51.3%), SCC for 50/191 (26.2%), and concurrent BCC + SCC for 43/191 (22.5%); 710/901 (78.8%) had no skin malignancy. No statistically significant differences emerged in the distribution of BCC, SCC, and combined tumors across organ groups (*χ* ^2^ test, *p* > 0.05). The graphical distribution of tumor types is shown in (Figure 1).

Among 901 recipients, exposure to CNI occurred in 796 (88.3%), antimetabolites in 516 (57.3%), mTOR inhibitors in 87 (9.7%), and maintenance corticosteroids in 609 (67.6%). Crude annualized incidence rates ranged between 1.88 and 2.68 per 100 person‐years across drug classes and agents (Table 2). At the agent level, NMSC occurred in 19.2% of tacrolimus‐exposed (120/626) versus 30.6% of cyclosporine‐exposed (53/174), and in 18.8% of MMF‐exposed (92/490) versus 38.5% of azathioprine‐exposed (10/26). Because combinations and sequential switches are common, these comparisons are non‐mutually exclusive and potentially confounded by transplant era and exposure duration; mutually exclusive crude contrasts showed higher risk with cyclosporine‐only versus tacrolimus‐only (30.6% vs. 19.1%; *χ* ^2^ *p* = 0.0013) and with azathioprine‐only versus MMF‐only (38.5% vs. 18.8%; *χ* ^2^ *p* = 0.0140).

**TABLE 2:** Annualized incidence rates of non‐melanoma skin cancer (NMSC) according to immunosuppressive drug class and individual agents (ever‐use).

Category/agent	Events (*n*)	Person‐years (PY)	Rate (per 100 PY)
**Immunosuppressive classes**			
Calcineurin inhibitors (CNI)	172	8007	2.15
Antimetabolites	102	5125	2.01
mTOR inhibitors	17	681	2.50
Maintenance corticosteroids	131	5809	2.26
**Individual agents**			
Tacrolimus	120	5317	2.26
Cyclosporine	53	2726	1.94
Mycophenolate mofetil (MMF)	92	4550	2.03
Azathioprine	10	585	1.88
Everolimus	13	532	2.44
Sirolimus	4	149	2.68

*Note:* Rates are expressed as events per 100 person‐years (PY), with PY calculated using total time on immunosuppression for each exposed patient. Ever‐use reflects any exposure during follow‐up, allowing for switches and combinations. This table provides a descriptive comparison of crude NMSC incidence across drug categories and specific agents; no causal inferences should be drawn from these unadjusted rates.

### 3.4 NMSC Per Year

Considering ever‐use (combinations and switches allowed), crude incidence rates were 2.15 per 100 person‐years for calcineurin inhibitors (172 events over 8,007 PY), 2.01 per 100 PY for antimetabolites (103 events over 5,125 PY), 2.50 per 100 PY for mTOR inhibitors (17 events over 681 PY), and 2.26 per 100 PY for maintenance corticosteroids (131 events over 5,809 PY) (Table 2).

Because exposure groups reflect ever‐use with concurrent or sequential regimens, denominators are not mutually exclusive and may differ by calendar era and follow‐up length. These crude rates, therefore, serve as descriptive context; adjusted, time‐varying models provide the primary inference (Table 3).

**TABLE 3:** This table summarizes the main statistical analyses of NMSC risk in transplant recipients.

NMSC frequency analysis by transplant type^#^				
	Total patients	NMSC cases	NMSC incidence (%)	*p* Value
Kidney	518	122	23.55%	0.001
Heart	121	31	25.62%	0.028
Liver	104	24	23.08%	0.187
HSCT	81	8	9.88%	0.080
Lung	54	3	5.56%	0.028
Kidney and Pancreas	12	1	8.33%	0.646
Liver (OLT) and kidney	7	1	14.29%	1.000
Heart and kidney	2	1	50.00%	0.780
Heart and Liver	2	0	0.00%	1.000

Cox regression by therapy (univariable)			
	HR	95% CI	*p* value
AZA	0.54	0.26–1.12	0.096
MMF	0.89	0.67–1.18	0.413
TAC	1.19	0.87–1.61	0.272
CsA	0.83	0.60–1.15	0.257
EVR	1.16	0.66–2.04	0.615
SRL	1.42	0.52–3.83	0.492
PDN	1.09	0.80–1.49	0.565

Cox regression by therapy (multivariable)			
	HR	95% CI	*p* Value
Age at first transplant	1.07	1.06–1.09	< 0.001
Sex	1.26	0.91–1.75	0.158
Heart^*^	1.13	0.71–1.79	0.619
Liver^*^	0.89	0.42–1.91	0.768
Lung^*^	0.62	0.19–1.97	0.416
HSCT^*^	1.13	0.39–3.27	0.819
Multi‐organ^*^	0.68	0.21–2.19	0.520
AZA	0.66	0.29–1.51	0.320
MMF	0.86	0.61–1.21	0.390
TAC	0.96	0.50–1.86	0.904
CsA	0.72	0.37–1.39	0.325
EVR	1.01	0.54–1.91	0.967
SRL	0.94	0.31–2.83	0.910
PDN	1.02	0.69–1.49	0.937

*Note:* The survival analysis panel shows the log‐rank test for NMSC‐free survival comparing heart versus kidney transplants and the global comparison across transplant types. The “Cox regression analysis (univariable)” panel reports hazard ratios (HRs) with 95% confidence intervals (CI) and *p* values for key individual predictors (age at first transplant, time since transplant, current age, sex, and ever‐use of each immunosuppressive agent), without mutual adjustment. The “Cox regression by therapy (multivariable)” panel shows the fully adjusted model including age at first transplant, sex, transplant group (Groups 1–4 vs. Group 5, kidney), and ever‐use of each agent, yielding adjusted HRs with 95% CIs and *p* values. The upper panel reports crude NMSC frequencies by transplant type, with incidence proportions and *p* values from *χ* ^2^ tests comparing each category with the overall distribution.

Abbreviations: AZA = azathioprine, CI = confidence interval, CsA = cyclosporine, EVR = everolimus, HR = hazard ratio, HSCT = hematopoietic stem‐cell transplantation, MMF = mycophenolate mofetil, OLT = orthotopic liver transplant, PDN = prednisone, ref = reference category, SRL = sirolimus, TAC = tacrolimus.

^#^ *p* values in the NMSC frequency panel are derived from χ^2^ tests comparing each transplant type with the remainder of the cohort.

* ref: Kidney.

### 3.5 Kaplan–Meier Survival Analysis

Survival analysis stratified by transplant type showed variable NMSC‐free survival probabilities across different transplant categories. Although no statistically significant difference was observed among the groups (all group‐vs.‐others log‐rank tests were non‐significant), the survival curves suggest a trend toward earlier NMSC onset in kidney and HSCT recipients compared to heart or lung transplant recipients.

**FIGURE 2:**
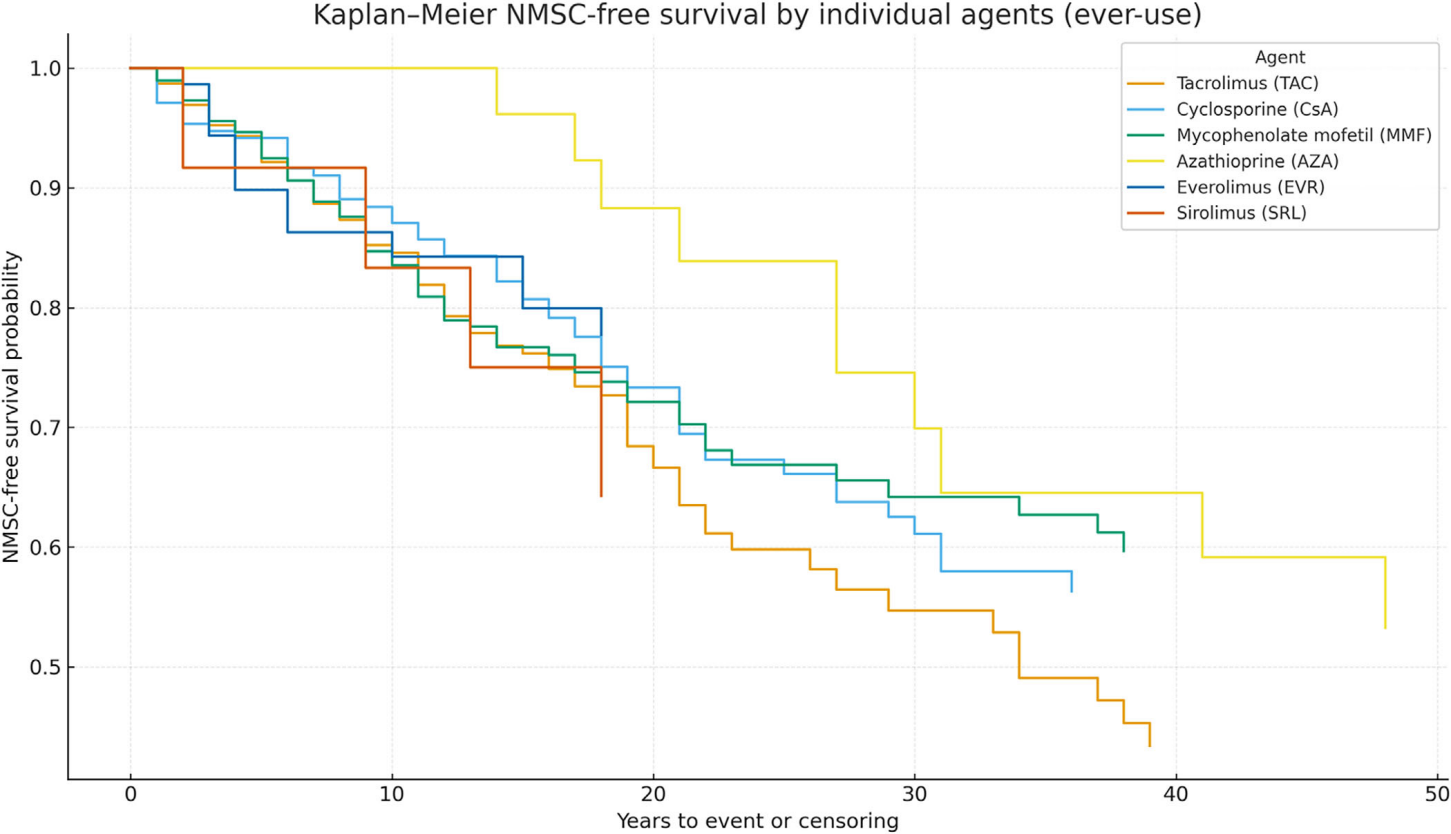
Kaplan–Meier estimates of NMSC‐free survival stratified by ever‐use of antimetabolite class (AZA/MMF/mTORi/none) and calcineurin inhibitor agent (TAC/CsA). Groups are ever‐use and therefore non‐mutually exclusive; curves are descriptive. Adjusted comparisons are reported in Cox models (Table 3).

**FIGURE 3:**
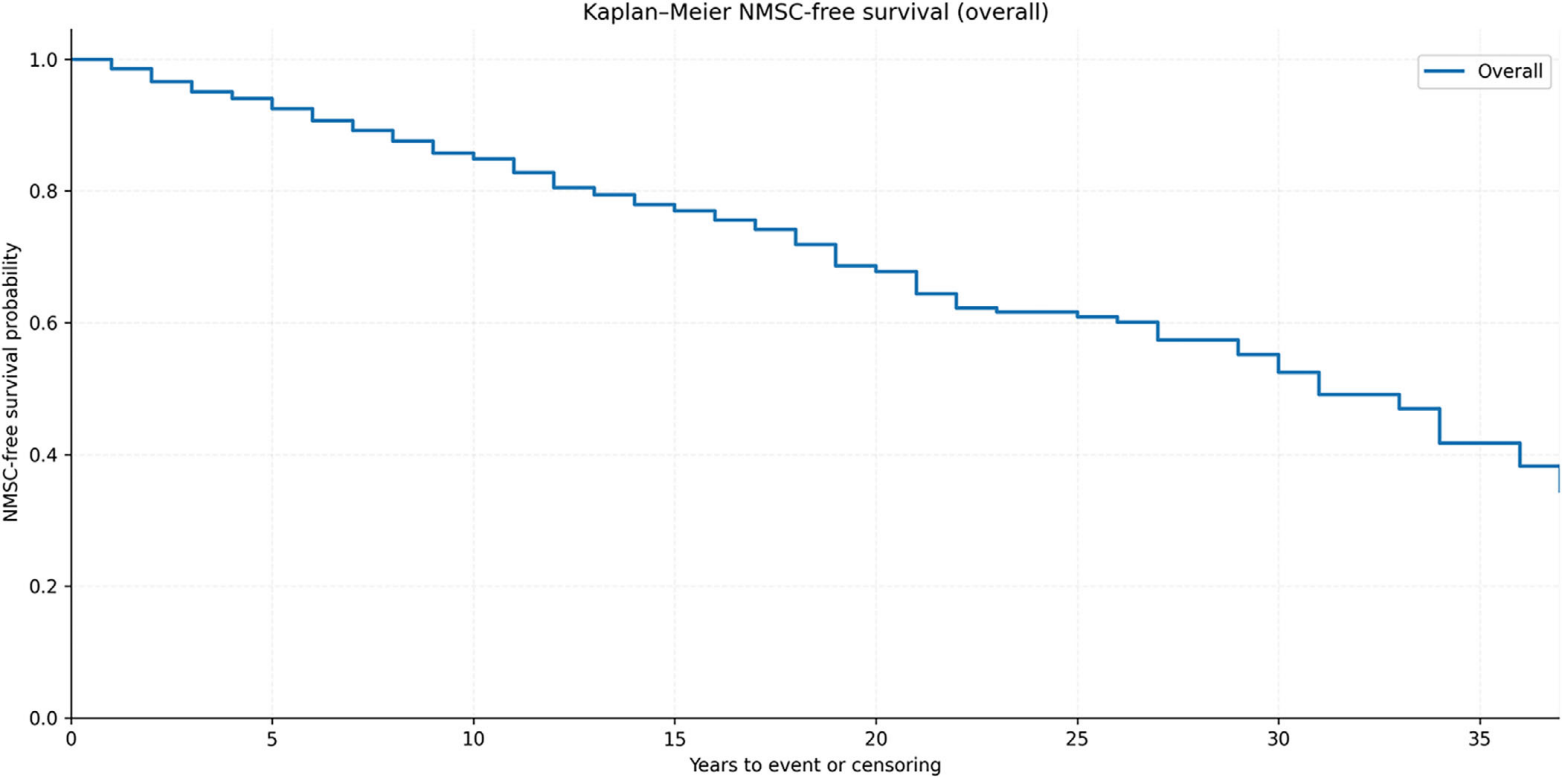
Kaplan–Meier estimates of overall NMSC‐free survival in the entire transplant cohort. The curve is truncated at 37 years (*n* at risk < 10 beyond this time point) to avoid unstable tail estimates. The endpoint was time from first transplant to first histologically confirmed NMSC; patients without NMSC were censored at last dermatologic follow‐up.

The median NMSC‐free survival time was comparable across groups; Kaplan–Meier curves did not show significant differences in NMSC‐free survival across transplant groups (log‐rank *p* > 0.05). Kaplan–Meier analyses were also evaluated for the antimetabolite category (AZA vs. MMF vs. mTOR inhibitors vs. none) and the calcineurin inhibitor agent (tacrolimus vs. cyclosporine), which defined the comparison groups. Log‐rank testing showed no statistically significant separations between antimetabolite groups or between CNI agents; curves suggested earlier events among AZA users relative to MMF/mTORi, and broadly similar trajectories for tacrolimus and cyclosporine. Corresponding curves are presented in (Figure 2). Overall NMSC‐free survival for the entire cohort is shown in Figure 2A; the curve is truncated at 37 years (*n* at risk < 10) to avoid unstable tail estimates (Figure 3).

### 3.6 Cox Regression Analysis

In Cox models, older age at first transplant was independently associated with higher NMSC risk. Organ type and drug class/agent ever‐use were not independently associated after adjustment; for antimetabolites, point estimates suggested higher hazard with AZA and lower with MMF, but confidence intervals crossed 1 (limited AZA exposure). Tacrolimus versus cyclosporine showed no material difference after accounting for era. mTOR inhibitors were neutral to modest and non‐significant. Prednisone proxies yielded attenuated, non‐significant estimates (Table 2).

## 4 Discussion

In this long‐term cohort, variation in NMSC risk was driven primarily by host factors rather than by the transplanted organ itself. Older age at first transplant was the strongest independent predictor of NMSC: each additional year of age at transplantation conferred an ∼7% increase in hazard (adjusted HR 1.07, 95% CI 1.06–1.09), corresponding to an almost two‐fold higher risk for patients transplanted 10 years later.

Kidney and heart transplant recipients were the most represented groups, reflecting national transplant distributions. These recipients also accounted for the majority of NMSC cases, in line with their known elevated risk due to prolonged exposure to CNIs and more intensive regimens, particularly in heart transplants.

Conversely, liver and HSCT recipients showed lower overall NMSC incidence in our cohort, which may reflect less aggressive or shorter‐duration maintenance immunosuppressive regimens and, for some liver recipients, tapering or discontinuation of therapy in long‐term survivors. However, the HSCT subgroup was relatively small and characterized by shorter and more heterogeneous follow‐up, so organ‐specific differences in NMSC risk, particularly for HSCT, should be interpreted with caution and regarded as exploratory.

Despite variation in absolute tumor counts, the proportional distribution of BCC, SCC, and combined BCC + SCC did not differ by organ (*χ* ^2^ = 9.92, *p* = 0.447), indicating a comparable subtype mix across transplant categories. When analyses were reframed by immunosuppressive therapy, Kaplan–Meier curves stratified by the specific agents showed no significant separations. In the multivariable analysis, age at first transplant was the principal predictor [25, 26, 27]; AZA exposure was non‐significant due to the small group number, whereas other drugs displayed broadly comparable hazards after accounting for era. Taken together, these findings refine prior reports attributing elevated NMSC risk to CNI exposure per se, suggesting that historical signals may partly reflect era effects, AZA use, and cumulative exposure, rather than a uniform class effect of CNIs [28, 29].

Regarding the relative risk of different NMSC subtypes (SCC vs. BCC), they did not significantly vary among transplant types in our cohort (*χ* ^2^ = 9.92, *p* = 0.447). This contrasts with prior studies reporting a higher SCC‐to‐BCC ratio in heart and lung transplant recipients [30], possibly due to differences in immunosuppressive protocols or cumulative UV exposure in our population; one possible explanation lies in the variability in follow‐up duration across transplant groups, as SCCs typically develop after longer periods of immunosuppression than BCCs. Additionally, differences in the prevalence of specific immunosuppressive regimens among transplant types may have influenced these patterns. Despite the long calendar span of the dataset, the relatively short mean follow‐up time per patient may have limited the power of this analysis to detect subtype‐specific trends.

To contextualize these subtype patterns by ambient UV, Australian OTR cohorts, largely of fair phototype, comparable to European‐descended populations, report very high cumulative NMSC incidences (≈7% at 1 year, 45% at 10 years, 82% at 20 years) with a pronounced SCC burden, consistent with intense UV exposure and historical regimen mix [31, 32]. By contrast, lower‐UV European cohorts show elevated risks relative to the background population but generally lower absolute burdens and different SCC:BCC balances: in the Netherlands, renal‐transplant recipients had an ≈250‐fold increase in SCC and 10‐fold in BCC compared the general population. In Sweden, BCC risk was strikingly increased, but a lower SCC:BCC ratio was noted—likely reflecting latitude and follow‐up duration—supporting the role of cumulative UV and exposure time in shaping subtype distributions [33].

These cross‐setting contrasts reinforce that ambient UV dose, together with cumulative immunosuppression and agent‐specific exposure, is a key determinant of both overall NMSC burden and the SCC:BCC mix in transplant recipients [31]. Still within our groups, older age at first transplant also significantly increased NMSC risk, likely reflecting the role of accumulated UV damage and immunosenescence in these patients, while those transplanted at a younger age may represent a subset with inherently lower oncologic risk [14, 34].

Also, our data nuance reports that linked CNI‐based regimens to substantially higher NMSC risk (e.g., via impaired DNA repair, UV‐induced mutagenesis, and dampened antitumor surveillance) [10, 14, 16, 18]; in our cohort, the CNI signal largely attenuated once era effects and agent‐specific exposure were accounted for. Residual confounding (UV dose, surveillance intensity) and limited power for smaller subgroups (e.g., AZA, SRL) should be remembered, but the overall pattern supports exposure intensity and agent‐specific effects over a uniform class effect of CNIs [19].

A possible explanation lies in the evolution of immunosuppressive protocols. In particular, maintenance corticosteroid dosing has decreased substantially over time, with more recent protocols favoring lower doses and shorter courses, which may have attenuated any independent association between “prednisone ever‐use” and NMSC risk in our analyses. Moreover, CNI minimization strategies have gained ground, aiming to balance graft protection with reduced long‐term oncologic risk. In line with this trend, our institution adopts a conservative approach, favoring the lowest effective immunosuppressive doses. Moreover, the variability in combinations and dosage within the therapy groups may have diluted any potential association between regimen type and NMSC risk [3, 14, 20, 22, 35, 36, 37, 38].

Altogether, these findings emphasize the need to move beyond simplistic classifications based on therapy type and instead focus on cumulative immunosuppressive exposure, transplant age, and individual risk factors to refine dermatological surveillance in transplant populations.

### 4.1 Strengths and Limitations

The retrospective design may introduce selection bias and data gaps (especially exposure granularity, which is imperfect—dose/intensity, tapering, precise timing of switches, and complete data on depleting induction were not uniformly available). Individual UV metrics and humoral, molecular, and genetic data were not captured, so age at transplant has also served as a proxy for cumulative photodamage. Event counts in smaller subgroups (notably AZA and SRL) widen confidence intervals and temper inference; evolving detection and management over decades may also influence observed risks. Finally, death was not modeled as a competing risk, which could bias subtype‐specific incidence in long‐term survivors.

Within these bounds, the therapy‐centered, cumulative exposure approach strengthens the conclusion that long‐term immunosuppressive burden and agent‐specific effects, particularly AZA, better explain NMSC risk than organ category per se.

## 5 Conclusion

This retrospective study offers a comprehensive analysis of NMSC risk in transplant recipients, integrating transplant type, immunosuppressive exposure, and tumor subtypes. The overall crude incidence of NMSC was 21.2%, corresponding to an overall incidence rate of approximately 1.9 per 100 person‐years. Kidney and heart transplant recipients exhibited the highest crude NMSC frequencies, whereas lung and HSCT recipients showed the lowest, in keeping with differences in immunosuppressive intensity and follow‐up duration.

Across analyses centered on immunosuppression, age at first transplant consistently emerged as the main independent determinant of NMSC risk, whereas organ category and ever‐use of individual immunosuppressive agents were not independently associated after adjustment. Crude incidence rates increased steadily with time since transplant (cumulative immunosuppressive exposure), and Kaplan–Meier analyses by drug class and agent were broadly concordant with these findings.

Taken together, these results support individualized dermatologic surveillance prioritizing time since transplant (cumulative immunosuppression) and older age at first transplant, rather than nominal regimen or organ type. Future prospective studies incorporating quantitative UV exposure, dose/intensity metrics, and molecular profiling are warranted to refine risk stratification and optimize prevention in this vulnerable population.

## Author Contributions

**Alba Guglielmo, Michelangelo La Placa**: methodology, formal analysis, supervision, visualization, writing – review and editing. **Corrado Zengarini**: conceptualization, software, project administration, formal analysis, writing – original draft, writing – review and editing. **Vittoria Tagnin**: data curation, investigation. **Gioia Sorbi**: supervision. **Bianca Maria Piraccini**: supervision, visualization. **Alessandro Pileri**: conceptualization, funding acquisition, supervision, writing – review and editing. **Marco Pignatti**: conceptualization, supervision, writing – review and editing.

## Funding

This study was supported by Università di Bologna.

## Ethics Statement

The study was approved by the local ethical committee of Bologna (Approval No. Clin. Isto. Tp.19).

## Consent

The patients in this manuscript have given written informed consent to publish their case details.

## Conflicts of Interest

The authors declare no conflicts of interest.

## Use of Artificial Intelligence Statement

AI‐assisted tools (ChatGPT, OpenAI) were employed for language editing, text summarization, and improvement of the illustrated graphs, with all outputs reviewed, verified, and edited by the authors for accuracy.

## Data Availability

The data that support the findings of this study are available from the corresponding author upon reasonable request.
